# Picking transplant glomerulopathy out of the CAN: evidence from a clinico-pathological evaluation

**DOI:** 10.1186/1471-2369-13-128

**Published:** 2012-09-28

**Authors:** Qiquan Sun, Xianghua Huang, Song Jiang, Caihong Zeng, Zhihong Liu

**Affiliations:** 1 Research Institute of Nephrology, Jinling Hospital, Nanjing University School of Medicine, Nanjing, 210002, China

**Keywords:** Antibody-mediated injury, Chronic allograft nephropathy, Hepatitis C virus, Transplant glomerulopathy

## Abstract

**Background:**

Since the term chronic allograft nephropathy (CAN) was removed from the Banff scheme in 2005, transplant glomerulopathy (TG) has been regarded as a clinicopathological entity that is one of the major causes of graft loss. To assess the distinction between CAN and TG, we performed a comprehensive evaluation comparing TG with traditional CAN.

**Methods:**

We compared the clinicopathological features of 43 cases of TG with 43 matched cases of non-TG CAN (non-TG group) after renal transplantation. TG was diagnosed by light microscopy based on the double contours of the glomerular basement membranes, and the Banff 97 classification system was used to score TG severity (cg0-3).

**Results:**

Compared to the control group, we found a significantly higher incidence of positivity for human leukocyte antigen class-I and II antibodies, a higher incidence of hepatitis C virus (HCV) infection, and poorer graft survival in TG patients. Clinically, TG was associated with a higher prevalence of proteinuria, hematuria, anaemia and hypoalbuminemia. Histologically, TG strongly correlated with antibody related microcirculatory injuries, including glomerulitis, peritubular capillaritis and peritubular capillary (PTC) C4d deposition. Interestingly, the TG patients showed a significantly higher incidence of IgA deposition than the control patients. C4d-positive TG was correlated with higher TG and PTC scores, and PTC C4d deposition was correlated with a more rapid progression to graft dysfunction. TG accompanied by HCV infection was associated with heavier proteinuria, higher TG and C4d scores, and poorer graft survival.

**Conclusions:**

TG presents clinicopathological features that are distinct from non-TG cases and leads to poorer outcomes. PTC C4d deposition is related to a more rapid progression to graft loss, suggesting ongoing antibody reactivity. HCV-positive TG is a more severe sub-entity, that requires further investigation.

## Background

Transplant glomerulopathy (TG), a pathologic condition of renal allografts that was first described 40 years ago, was considered to be a feature of chronic allograft nephropathy (CAN) for decades. However, emerging evidence suggests that TG is a pathologic entity that is distinct from other forms of CAN. Since the elimination of the term CAN [1] by the Banff working group in 2005, TG has been recognised as a lesion with a specific morphology showing a strong association with immune mechanisms [2]. The characteristic thickened capillary walls and double contours of the glomerular basement membrane (GBM) after periodic-acid-Schiff and/or silver staining can be observed using light microscopy. Clinically, TG is characterised by proteinuria, hypertension and declining graft function. In addition, TG is often associated with indicators of antibody-mediated rejection (AMR) such as the presence of donor-specific antibodies (DSAs) and positive peritubular capillary (PTC) C4d staining, and it is therefore considered to be an example of chronic AMR.

Previous studies have demonstrated that TG is strongly associated with circulating alloantibodies [3,4], positive PTC C4d staining [5], and prior AMR [6]. These findings substantiate the hypothesis that humoral immunity plays a key role in the pathogenesis of TG. However, PTC C4d deposition is not necessary for diagnosis of TG [7], and the significance of PTC C4d deposition in TG remains unclear, as the data reported from different studies are highly variable [7,8].

Recent studies investigating kidney transplant failure have focused on the role of humoral rejection. A number of the observed lesions are considered to represent antibody-mediated injury, such as PTC C4d deposition, glomerulitis, peritubular capillaritis, PTC basement membrane multi-layering, and even TG. Interestingly, lesions such as antibody-mediated microcirculation injuries were recently reported to be the major causes of late kidney transplant failure [9]. As TG is believed to be a result of antibody reactivity, it will be interesting to determine the relationship between TG and other antibody-mediated microcirculatory injuries.

In this study we compare the clinicopathological features of TG patients with traditional CAN patients. Our data show that TG strongly correlates with antibody-mediated microcirculation injury. Furthermore, PTC C4d deposition is associated with a more rapid progression to allograft dysfunction, and we propose that this is due to ongoing antibody reactivity. We also describe, for the first time, IgA deposition in the mesangial area of TG patients. We further propose that hepatitis C virus (HCV)-positive TG is a more severe sub-entity that requires further investigation.

## Methods

### Patient selection and evaluation

From January 2004 to December 2008, 327 renal allograft biopsies were performed at our institute. The biopsies were performed to investigate worsening renal function and/or proteinuria. Among these cases, 43 patients had been diagnosed with TG based on pathological duplication of the GBM, after exclusion of other conditions that might result in similar histological changes, such as recurrent membranoproliferative glomerulonephritis and thrombotic micro-angiopathies.

The TG patients were matched with 43 cases of non-TG traditional CAN (non-TG group) as controls for post-transplantation observation. The selection of the control group was based on the following criteria: (1) transplantation was performed in the same period; (2) non-TG traditional CAN was confirmed by biopsy; (3) no duplication of the GBM was observed; and (4) patients were followed-up with for more than a year after the biopsy was performed. Patients who suffered from acute rejection at the time of biopsy were excluded from both groups. Written informed consent was obtained from all patients. The Human Subjects Committee of Jinling Hospital, Nanjing University School of Medicine approved all study protocols.

An electronic patient follow-up system has been used to record information at each follow-up appointment since 2000. Clinical and laboratory data on the patients were extracted from their medical records, including their age, gender, primary disease, post-transplantation day at biopsy, serum levels of creatinine and albumin, proteinuria, hematuria, and HCV infection status at biopsy, type of immunosuppressive drugs used, history of biopsy-proven acute rejection episodes and graft outcome.

### Immunosuppressants

Two main immunosuppressive protocols were employed: 1) cyclosporine A (CsA), mycophenolate mofetil (MMF) and steroids; and 2) Tacrolimus (Tac), MMF and steroids. The initial dose of MMF was 1.5 g/day. The maintenance doses were adjusted to trough levels of 6–12 ng/ml during the first 6 months and 4–8 ng/ml during the second 6 months for Tac, or 150–250 ng/ml during the first 6 months and 100–200 ng/ml during the second 6 months for CsA. The maintenance dose of corticosteroid was 5 mg/day 1 year after transplantation. Initially, some patients were given azathioprine instead of MMF.

### Histopathology

The biopsies were evaluated via light microscopy, immunofluorescence (IF) and electron microscopy. IF microscopy was employed for the detection of IgG, IgM, IgA, C3, C4, and C1q (DAKO, Denmark). C4d staining was routinely performed using an indirect IF technique in frozen sections with a primary affinity-purified monoclonal mouse anti-human antibody (dilution 1:50; 1.5 hours incubation at room temperature; Quidel San Diego, CA) and a FITC-labelled affinity-purified secondary rabbit anti-mouse IgG antibody (1:20; 40-minutes incubation at room temperature; DAKO, Denmark). Staining was performed according to standard procedures. All of the biopsies contained at least 10 glomerular and 2 arterial sections. The biopsies were diagnosed and scored according to the Banff classification [10]. TG was diagnosed via light microscopy based on double contours of the GBM, supported by IF analyses, showing mesangial IgM and/or C3 or negative IF findings. The electron microscopy results also corresponded to a diagnosis of TG. The Banff 97 classification system was used to score TG severity (cg0-3). PTC inflammation was scored based on the Banff 2007 classification system [11].

To assess cellular infiltration in graft tissues, 2 μm sections were deparaffinised with xylene and rehydrated using graded ethanol concentrations. Antigen retrieval was performed by applying steam for 5 minutes (a heat-induced epitope retrieval method). Immunohistochemical staining was conducted using the avidin-biotin- peroxidase technique employing 3,3-diaminobenzidine as the chromogen with the automated BioTek system (Ventana/BioTek Solutions, Tucson, AZ). All stainings were performed using rabbit anti-mouse IgG (Dako, Trappes, France) at a 1:100 dilution. CD3, CD4, and CD8 were employed as markers for T lymphocytes, and CD68 was used as a marker for macrophages (all antibodies were obtained from Dako).

Several histopathological parameters were studied, including transplant glomerulopathy, glomerulitis, peritubular capillaritis and mesangial matrix (MM) expansion. Interstitial fibrosis (ci), tubular atrophy (ct), and fibrous intimal thickening (cv) were used to characterise chronic lesions, and all of the lesions were scored based on the Banff 1997 and/or Banff 2007 classification systems.

### Anti-HLA antibody analysis

Patients were screened for human leukocyte antigen (HLA) class I and class II antibodies using FlowPRA® (One Lambda, USA) at the time of biopsy. Sera showing >10% FlowPRA® class I and/or II reactivity were considered anti-HLA antibody-positive. All of the patients PRA test were negative prior to renal transplantation. Additional sera were stored for future use.

### Statistical analysis

Measurement data were expressed as the means ± SD. Differences between groups were analysed with the Student’*t* test, and the Student-Newman-Keuls method was used for multiple comparisons. Qualitative data were described as percentages and analysed using the Chi-square (χ^2^) test as indicated. Survival curves were calculated via the Kaplan-Meier survival analyses and compared using the log-rank test. The reported *P* value is two-sided, and the values less than 0.05 were considered statistically significant. All analyses were performed using SPSS software (Version 13.0, SPSS Inc., USA).

## Results

### Clinical findings

The demographic and clinical details of the two groups are outlined in Table 1. There were no significant differences between the demographic characteristics of the groups. Allograft biopsies were performed at 4.93 ± 2.72 and 4.53 ± 2.52 years post-transplantation in the non-TG and TG groups, respectively. All of the patients were on triple immunosuppressive medications with calcineurin inhibitors, MMF, and prednisone.

**Table 1:** **Demographic characteristics of thestudy population**

**Patient characteristics**	**TG (n = 43)**	**non-TG (n = 43)**	***P* value**
Mean age (years)	42.88 ± 10.99	41.81 ± 8.91	0.356
Male (%)	34 (79.1%)	29 (67.4%)	0.330
Primary disease (%)			
CGN	31 (72.1%)	35 (81.4%)	0.444
DN	1 (2.3%)	0	1.0
IgAN	2 (4.7%)	2 (4.7%)	1.0
FSGS	2 (4.7%)	1 (3.3%)	1.0
Alport syndrome	0	2 (4.7%)	0.494
Others	7 (16.3%)	3 (7.0%)	0.313
Time from transplant to biopsy (years)	4.93 ± 2.72	4.53 ± 2.52	0.772
Immunosuppressive regimen (%)			
CMP	20 (46.5%)	26 (60.5%)	0.28
FMP	19 (44.2%)	13 (30.2%)	0.265
CAP	3 (7.0%)	4 (9.3%)	1.0
Other	1 (2.3%)	0	1.0

Data are presented as mean ± SD or number of cases. CGN: chronic glomerulonephritis; DN: diabetic nephropathy; IgAN: IgA nephropathy; FSGS: focal segmental glomerulosclerosis; CMP: cyclosporine A (CsA), mycophenolate mofetil (MMF) and steroids; FMP: Tacrolimus (Tac), MMF and steroids; CAP: CsA, azathioprine (AZA) and steroids.

TG was correlated with a higher incidence of proteinuria (40/43, 93.0% vs. 20/43, 46.5%, *P* < 0.001) and hematuria (13/43, 30.2% vs. 5/43, 11.6%, *P* = 0.034). Almost all of the patients in the TG group suffered from proteinuria, which was significantly heavier compared to the control group (2.01 ± 1.60 g/d vs. 0.89 ± 0.96 g/d, *P* = 0.001). In the TG group, the majority of the patients (30/43, 69.8%) had urine protein levels of over 1 g/day, whereas the percentage of patients showing such levels was only 25.5% (11/43) in the non-TG group (*P* < 0.001). TG was also correlated with higher incidences of anaemia (37/43, 86.0% vs. 28/43, 65.1%; *P* = 0.022) and hypoalbuminemia (26/43, 60.5% vs 5/43, 11.6%, *P* < 0.001). The mean haemoglobin level in the TG group was significantly lower than the non-TG group (9.48 ± 2.18 vs. 10.66 ± 2.01 *P* = 0.011). We also observed a higher prevalence of HCV infection in TG group than in the non-TG group (Table 2). Interestingly, graft function and previous acute rejection episodes were similar in the two groups.

**Table 2:** **Clinic features of theTG and non**-**TG groups at thetime of biopsy**

**Clinic features**	**TG (n = 43)**	**non-TG(n = 43)**	***P *value**
Proteinuria (g/24 h)	2.01 ± 1.60	0.89 ± 0.96	0.001
Patients with proteinuria	40 (93.0%)	20 (46.5%)	<0.001
<1 g/24 h	10 (23.3%)	9 (20.9%)	0.565
1-3.5 g/24 h	22 (51.2%)	10 (23.2%)	0.007
>3.5 g/24 h	8 (18.6%)	1 (2.3%)	0.014
Hematuria	13 (30.2%)	5 (11.6%)	0.034
Hemoglobin (g/dl)	9.48 ± 2.18	10.66 ± 2.01	0.011
Patients with anemia	37 (86.0%)	28 (65.1%)	0.022
Mild (9-12 g/dl)	17 (39.5%)	18 (41.9%)	0.500
Moderate (6-9 g/dl)	18 (41.9%)	10 (23.3%)	0.050
Severe (<6 g/dl)	2 (4.7%)	0	0.247
Albumin (g/L)	33.79 ± 4.89	39.50 ± 4.48	0.330
Hypoalbuminemia	26 (60.5%)	5 (11.6%)	<0.001
Creatinine (mg/dl)*	2.12 ± 1.26	2.00 ± 1.02	0.600
Patients with the history of rejection	15 (34.9%)	14 (32.6%)	0.943
HCV infection	15 (34.9%)	3 (7.0%)	0.003
HLA-I antibodies present	9/30(30%)	1/23 (4.3%)	0.018
HLA-II antibodies present	17/30 (56.7%)	4/23 (17.4%)	0.004

Data are presented as mean ± SD or number of cases. *Creatinine compared in TG group had excluded 3 dialysis patients.

### Anti-HLA antibodies

An anti-HLA antibody test was performed at the time of the biopsy in 30 patients with TG and in 23 patients with non-TG, and significantly more patients in the TG group were classed as positive (>10% FlowPRA® class I and/or II reactivity) for anti-HLA antibodies (20/30, 66.7% vs. 5/23, 21.7%; *P* = 0.001). Among these patients, 15% were positive for class I, 55% for class II and 30% for both class I and class II antibodies. The incidence of positivity was significantly higher in the TG group for both HLA-I and II antibodies.

### Pathologic features

All of the patients were subjected to an ultrastructural histological examination, and patients with a confounding diagnosis of immune complex glomerulonephritis were excluded. The diagnosis of TG was confirmed via electron microscopy. Compared to the non-TG group, TG was strongly correlated with hallmarks of antibody-mediated lesions (Table 3), such as peritubular capillaritis (40/43, 93.0% vs. 15/43, 34.9%; *P* < 0.0001), glomerulitis (40/43, 93.0% vs. 27/43, 62.8%; *P* = 0.001), and PTC C4d deposition (30/43, 69.8% vs. 14/43; 32.6%, *P* = 0.001). In addition, the samples from the TG group received significantly higher scores of C4d (1.91 ± 1.32 vs. 0.58 ± 0.96; *P* < 0.0001), PTC (1.88 ± 0.79 vs. 0.49 ± 0.73; *P* < 0.0001), glomerulitis (1.35 ± 0.69 vs. 0.79 ± 0.74; *P* < 0.001) and MM expansion (1.58 ± 0.62 vs. 1.07 ± 0.63, *P* < 0.001). Interestingly, we also noted a higher incidence of IgA staining in the TG group (44.2% vs 16.3%; *P* = 0.005). The incidences and degrees of chronic tubular, interstitial, and vascular sclerosis were similar between the TG and non-TG groups. In the case of glomerulitis, the majority of infiltrating cells were CD68+, with the next most prevalent groups being CD4+ and CD8+ cells. CD4+ cells were significantly more abundant in the samples from the TG group, compared to the non-TG group (*P* = 0.002). Similarly, labelling the PTC via CD31 staining revealed that CD68+ cells were also the major cause of PTC inflammation (data not shown).

**Table 3:** **Pathologic features of theTG and non**-TG groups*

**Pathology features**	**TG** (**n** = **43**)	**non**-**TG** (**n** = **43**)	***P *value**
TG score	1.88 ± 0.85	0.0 ± 0.0	<0.0001
C4d+	30 (69.8%)	14 (32.6%)	0.001
C4d score	1.91 ± 1.32	0.58 ± 0.96	<0.0001
Peritubular capillaritis present	40 (93.0%)	15 (34.9%)	<0.0001
PTC score	1.88 ± 0.79	0.49 ± 0.73	<0.0001
Glomerulitis present	40 (93.0%)	27 (62.8%)	0.001
Glomerulitis score	1.35 ± 0.69	0.79 ± 0.74	<0.001
Mesangial matrix expansion score	1.58 ± 0.62	1.07 ± 0.63	<0.001
Interstitial fibrosis (ci)	1.51 ± 0.83	1.33 ± 0.72	0.27
Tubular atrophy (ct)	1.49 ± 0.88	1.47 ± 0.77	0.89
Fibrous intimal thickening (cv)	1.14 ± 0.83	0.91 ± 0.68	0.16
IgA stain positive(%)	19 (44.2%)	7 (16.3%)	0.005
IgA score	0.70 ± 0.86	0.26 ± 0.62	0.008
CD4 cell infiltration in glomerular	0.82 ± 0.84	0.31 ± 0.53	0.002
CD8 cell infiltration in glomerular	0.86 ± 1.15	0.49 ± 0.90	0.127
CD68 cell infiltration in glomerular	7.84 ± 6.19	1.64 ± 2.98	<0.001

Data are presented as mean ± SD or number of cases. *Evaluation of the biopsies was based on the Banff ’07 acute and chronic indices, including presence of glomerulitis, TG, mesangial matrix expansion (based on silver stain), degree of PTC congestion, extent of interstitial fibrosis (based on trichrome stain) and tubular atrophy, vascular pathology, and deposition of C4d.

### Graft survival

**Figure 1:**
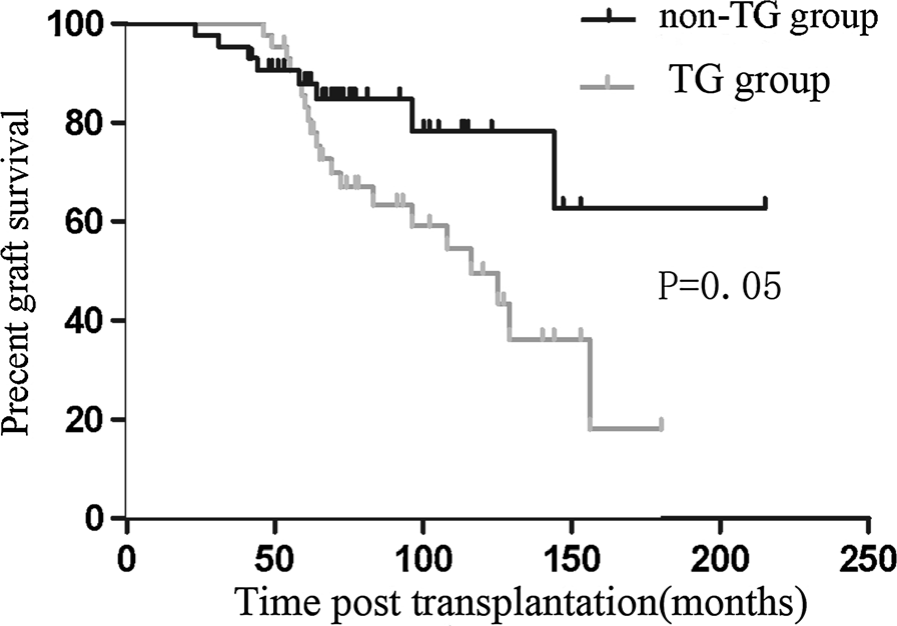
**The graft survival aftertransplantation of TG andnon**-**TG group.** The median graft survival was 115.4 months in TG group, while it was 170.4 months in non-TG group (*P* =0.05).

**Figure 2:**
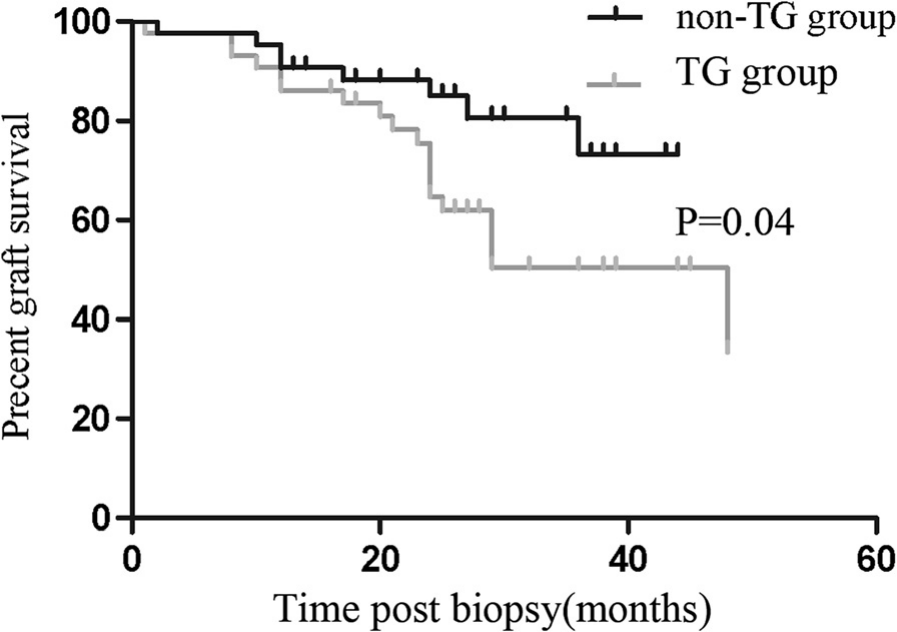
**The graft survival afterbiopsy of TG andnon**-**TG group.** The median graft survival after biopsy in TG group and non-TG was 34.1 months and 38.1 months respectively (*P* = 0.04).

Figure 1 shows the Kaplan-Meier survival curves for death-censored graft outcomes in the two groups. Significantly more patients in the TG group lost their grafts (44.2% vs. 18.6%; *P* = 0.05) during the follow-up period. The median graft survival was 115.4 months in the TG group, compared to 170.4 months in the non-TG group. Graft survival was also worse in the TG group when measured from the time of biopsy (34.1 months vs. 38.1 months; *P* = 0.04) (Figure 2).

### PTC C4d deposition in TG

Our data revealed a high incidence of PTC C4d deposition in samples from the TG group; however, PTC C4d deposition is not necessary for diagnosis of TG. Tables 4 and 5 allow comparison of the clinical and histological features between the C4d-positive (n = 30) and C4d-negative (n = 13) TG patients. C4d-positive TG was associated with a higher TG score (2.07 ± 0.89 vs. 1.44 ± 0.66, *P* = 0.018) and PTC score (2.10 ± 0.48 vs. 1.38 ± 1.12; *P* = 0.044). Significantly more individuals were positive for HLA-II antibodies among the C4d + TG patients vs. C4d- TG patients (14/19, 73.6% vs. 3/11, 27.3%; *P* = 0.018). Although there were no statistically significant differences detected, we observed a trend towards increased hypoalbuminemia and a higher incidence of HCV infection in the C4d + group.

**Table 4:** **Clinical features of theC4d**- **and C4d** + **in TG at thetime of biopsy**

**Clinic features**	**C4d** + **(n** = **30)**	**C4d**- **(n** = **13)**	***P *value**
Hematuria	9 (30%)	4 (30.8%)	0.487
Proteinuria (g/24 hrs)	2.44 ± 2.23	1.71 ± 1.49	0.218
Albumin(g/L)	33.86 ± 5.08	35.86 ± 2.25	0.020
hypoalbuminemia	21 (70.0%)	5 (38.4%)	0.055
Creatinine(mg/dl)	2.53 ± 1.83	2.61 ± 1.51	0.891
History of AR	10 (33.3%)	5 (38.5%)	0.504
HCV infection	13 (43.3%)	2 (15.4%)	0.108
HLA-I antibodies present	7/19 (36.8%)	2/11 (18.2%)	0.258
HLA-II antibodies present	14/19 (73.6%)	3/11 (27.3%)	0.018

Data are presented as mean ± SD or number of cases.

**Table 5:** **Pathologic features of theC4d**- **and C4d** + **in TG**

**Pathology features**	**C4d** + **(n** = **30)**	**C4d**- **(n** = **13)**	***P *value**
TG score	2.07 ± 0.89	1.44 ± 0.66	0.018
PTC score	2.10 ± 0.48	1.38 ± 1.12	0.044
Glomerulitis score	1.37 ± 0.67	1.31 ± 0.75	0.809
Mesangial matrix expansion Score	1.70 ± 0.58	1.31 ± 0.63	0.070
Interstitial fibrosis (ci)	1.50 ± 0.77	1.54 ± 0.96	0.900
Tubular atrophy (ct)	1.47 ± 0.77	1.54 ± 1.12	0.837
Fibrous intimal thickening (cv)	1.30 ± 0.72	0.77 ± 1.13	0.104
IgA stain positive(%)	16 (43.3%)	3 (23.1%)	0.065
IgA score	0.80 ± 0.84	0.46 ± 0.87	0.253

Data are presented as mean ± SD or number of cases.

### HCV infection in TG

**Figure 3:**
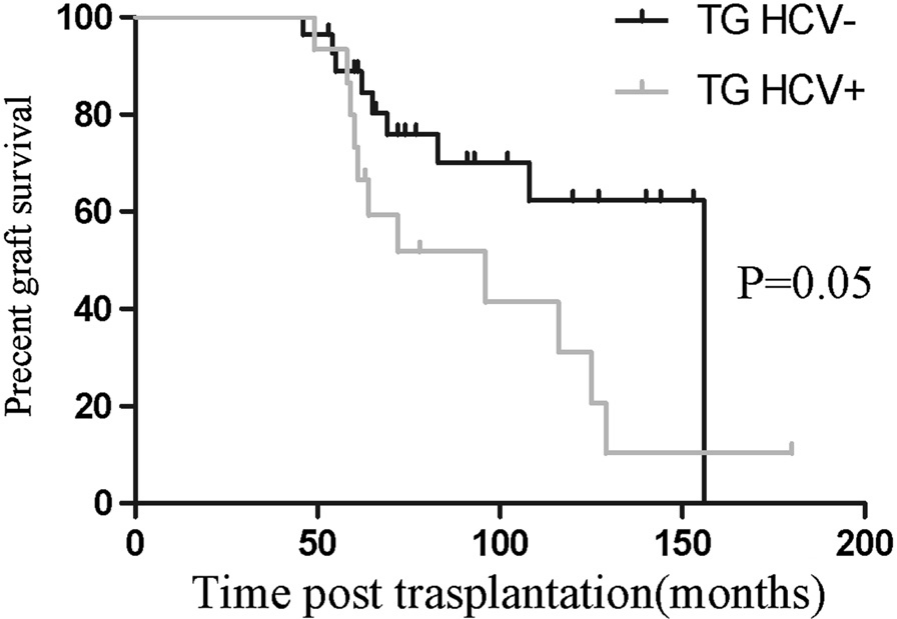
**The graft survival aftertransplantation of HCV**- **and HCV** + **in TG.** The median graft survival after transplantation in HCV- group and HCV + was 156 months and 96 months respectively (*P* = 0.05).

**Figure 4:**
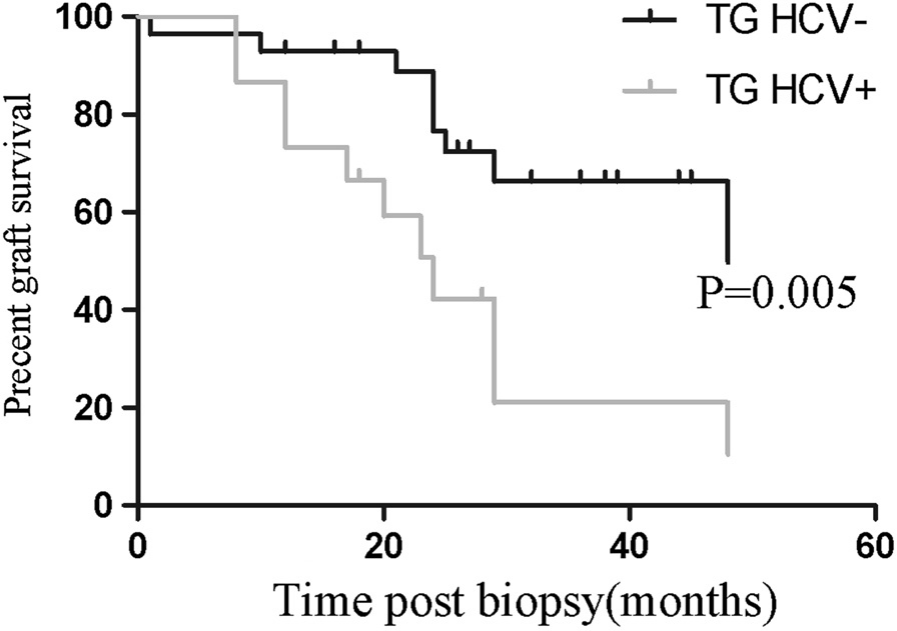
**The graft survival afterbiopsy of HCV** + **and HCV**- **in TG.** The median graft survival after biopsy in HCV- group and HCV + was 48 months and 24 months respectively (*P* = 0.005).

We observed that the incidence of HCV infection in the TG patients was significantly higher compared with the non-TG patients (34.9% vs. 7.0%; *P* = 0.003). As the characteristics of HCV-positive TG are not clear, we compared the clinical and pathologic features of HCV-positive and HCV-negative patients. The HCV-positive patients exhibited significantly heavier proteinuria (2.74 ± 1.71 vs. 1.65 ± 1.36; *P* = 0.029), lower serum albumin (31.15 ± 6.12 vs. 35.13 ± 3.28; *P* = 0.009) and poorer liver function. Pathologically, the HCV-positive samples received a higher C4d score (2.53 ± 1.06 vs. 1.57 ± 1.34, *P* = 0.021) and a higher TG score (2.27 ± 0.79 vs. 1.68 ± 0.81; *P* = 0.029) (Table 6), but the distribution of IgA staining did not differ between the HCV-positive and HCV-negative TG patients. In terms of graft survival (Figure 3 and 4), HCV-positive TG was associated with poorer graft survival. Furthermore, it is further notable that HCV-positive TG was correlated with a higher incidence of hematuria (46.7% vs. 21.4%; *P* = 0.087) and PTC C4d deposition (86.7% vs. 60.7%; *P* = 0.075).

**Table 6:** **Clinical and pathologic featuresof the HCV** + **and HCV**- **in TG**

**Clinical and Pathology features**	**HCV** + **(n** = **15)**	**HCV**- **(n** = **28)**	***P *value**
Hematuria	7 (46.7%)	6 (21.4%)	0.087
Proteinuria (g/24 hrs)	2.74 ± 1.71	1.65 ± 1.36	0.043
Serum albumin (g/L)	31.15 ± 6.12	35.13 ± 3.28	0.009
Elevated liver transaminases (%)	3 (20%)	0	0.037
Cryoglobulinemia present	2 (13.3%)	0	0.116
HCV RNA test positive	4 (26.7%)	0	0.011
TG score	2.27 ± 0.79	1.68 ± 0.81	0.029
C4d+	86.7% (13/15)	60.7% (17/28)	0.075
C4d score	2.53 ± 1.06	1.57 ± 1.34	0.021
IgA stain positive (%)	5 (33.3%)	14 (50%)	0.349

Data are presented as mean ± SD or number of cases.

## Discussion

In this report, we describe the clinicopathological features of patients with TG, C4d-positive TG and HCV-positive TG. Compared to the non-TG group, we find that: (1) TG is correlated with high incidences of proteinuria, anaemia and hypoalbuminemia and poorer graft survival; (2) TG is strongly correlated with antibody-mediated microcirculatory injury, especially in cases of glomerulitis and peritubular capillaritis; (3) TG accompanied by HCV infection is a more severe sub-entity and correlates with a poorer graft survival; and (4) TG is associated with a high incidence of IgA deposition in the mesangial area.

Our data show that TG patients present unique clinicopathological characteristics compared to non-TG patients. Clinically, TG was correlated with higher incidences of proteinuria, hematuria and hypoalbuminemia. While proteinuria was also observed in half of the non-TG patients, the amount of urine protein was significantly lower compared to the TG group. Similarly, anaemia was more common and severe in the TG group, and TG was associated with both reduced graft function and reduced graft survival [12]. Moreover, TG was correlated with the presence of anti-HLA antibodies, especially anti-HLA class-II antibodies [13]. Taken together these findings suggest that TG is a clinical entity that is distinct from non-TG cases. Notably, there was a significantly higher incidence of anaemia in the TG group, suggesting that other factors beyond the absence of TG are responsible for anaemia in TG patients.

**Figure 5:**
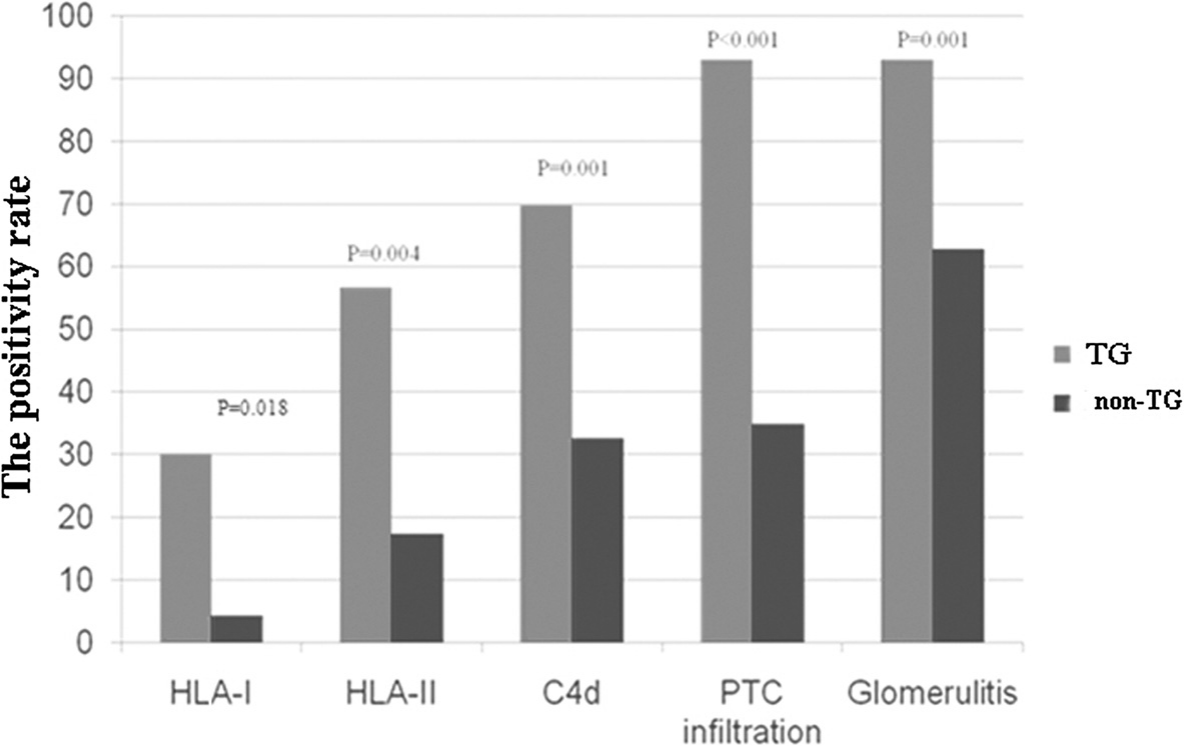
**TG is strongly correlatedwith the antibody andantibody**-**related lesions**: **the incidence of HLAantibodies (HLA**-**I antibodies**: **30%vs**. **4**.**3%**, **P** = **0**.**018**; **HLA**-**II antibodies**: **56**.**7%vs**. **17**.**4%**, **P** = **0**.**004),PTC C4d deposition(69**.**8%vs**. **32**.**6%**, **P** = **0**.**001),PTC inflammation(93**.**0%vs**. **34**.**9%**, **P** < **0**.**001)and glomerulitis(93**.**0%vs**. **62**.**8%**, **P** = **0**.**001)were higher in TGpatients**.

In addition to its clinical features, TG differs from non-TG cases with respect to histological features. Patients suffering from TG exhibited a higher incidence of peritubular capillaritis, glomerulitis, and PTC C4d deposition. These lesions, which are thought to be caused by antibody reactivity, have previously been described as antibody-mediated microcirculatory injuries [9]. Our data revealed a predominance of microcirculatory injuries in the TG group, supporting the hypothesis that TG is mediated by donor-specific antibodies. Almost all TG patients showed signs of glomerulitis, and/or peritubular capillaritis, and/or PTC C4d deposition (Figure 5). As the scores for chronic lesions, such as ci, cv and ct, were similar between the TG and non-TG groups, it is possible that the severe microcirculatory injuries observed in TG patients contribute to their poor graft outcomes.

While PTC C4d deposition has been regarded as a marker of *in situ* antibody reactivity [14], the reported incidences of PTC C4d deposition in TG patients are quite variable between different studies [15,16]. In the present cohort, C4d-positive TG is correlated with a higher incidence of positivity for HLA-II antibodies and lower levels of serum albumin. We did not find significant differences in other clinical features between cases of TG with or without PTC C4d deposition. Previous studies have shown that PTC C4d deposits are more common in TG than other glomerular diseases that occur after transplantation, and it has been suggested that PTC C4d deposits may indicate that *in situ* humoral rejection is relevant for the development of TG [17,18]. PTC C4d deposition has been shown to be correlated with circulating donor-specific antibodies, that induce endothelial cellular apoptosis independent of the action of the complement system [19]. Further data revealed that graft survival is significantly poorer in TG than in non-TG patients; more than 50% of patients diagnosed with TG achieved an end point of allograft loss or loss of >50% of the glomerular filtration rate over 36 months after transplantation [20]. Interestingly, C4d positivity was associated with a more rapid rate of transplant function decline [21,22]. Taken together, these findings suggest that PTC C4d deposition is very likely to be correlated with on-going alloantibody reactivity.

Our study revealed a higher incidence of PTC C4d staining, glomerulitis, and peritubular capillaritis compared to earlier reports. This finding is most likely due to a bias in patient selection; in the present cohort, all of the biopsies were for-cause biopsies, while earlier studies also included protocol biopsies [13]. Other possible reasons for these findings are that 1) after an acute antibody-mediated injury, the progression of capillary lesions does not involve complement activation; and 2) the interactions between antibodies and the endothelium are episodic and not continuous, making C4d an inconstant marker [4]. The cohort studies described here reflect an active process of TG, which may explain why patients showing PTC C4d deposition suffer from more rapid graft loss. Based on current evidence, we hypothesise that the development of TG is a long-term process, and that the associated doubled contour is most likely not (or not easily) reversible. However, active immunological responses to antibody reactivity might disappear, and we suggest that controlling active lesions after remission may slow graft dysfunction.

One-third of the TG patients included in this study suffered from HCV infection. This high incidence of HCV infection is concordant with previous studies [23,24]. As observed in the present study, HCV-positive TG is characterised by heavier proteinuria, lower serum albumin, higher TG and C4d scores, and poorer graft survival. These findings are supported by the results of Baid-Agrawal et al. [25], who demonstrated that graft survival in TG patients is poorer when they are HCV-positive. HCV infection may increase humoral immune alloreactivity in some patients, which is important for the development of TG. Based on the features described above, we thus conclude that HCV-positive TG is likely to be a more severe subtype of TG. It is possible that HCV infection can induce an aggressive process that leads to graft failure. As strong immunosuppressants usually stimulate the proliferation of HCV, these patients require careful observation.

The present study unexpectedly revealed a higher incidence of IgA deposition in the mesangial area in TG patients. To our knowledge, this is the first report describing an association of increased IgA deposition with TG. Because IgA deposition was similar in HCV-positive and HCV-negative patients, the increased incidence of glomerular IgA deposition in patients with TG appears to have no correlation with HCV infection. This interesting finding is supported by recently reported genome-wide analysis data showing a strong association between HLA and IgA nephropathy [26]. This study, identified three independent loci in the major histocompatibility complex, and the strongest HLA signal was identified in the region of HLA-DRB1 and HLA-DQB1. Another study further showed that the HLA region contains the strongest common susceptibility alleles that predispose patients to IgA nephropathy [27]. Taken together, these studies suggest a correlation between HLA antibodies and the development of IgA nephropathy, which may explain the high incidence of IgA deposition observed in our study. The role of HLA antibodies in IgA nephrology should be investigated in future studies.

## Conclusion

In conclusion, we demonstrate that TG patients exhibit significantly higher incidences of proteinuria, anaemia, hypoalbuminemia, positivity for HLA- II antibodies and HCV infection. TG is strongly correlated with antibody-mediated microcirculation injury, especially in cases of glomerulitis and peritubular capillaritis. The graft survival rate in TG patients is poorer compared with control patients. PTC C4d deposition is associated with a more rapid progression to allograft dysfunction, suggesting on-going antibody reactivity. HCV-positive TG is a more severe sub-entity that requires further investigation. Similarly, IgA deposition in the mesangial area was commonly found in TG patients, and determination of its role in TG requires further study.

## Abbreviations

AMR: Antigen-mediated rejection; CAN: Cronic allograft nephropathy; CsA: Cyclosporine A; cv: Fibrous intimal thickening; GBM: Glomerular basement membrane; HCV: Hepatitis C virus; HLA: Human leukocyte antigen; IF: Immunofluorescence; ci: Interstitial fibrosis; MM: Mesangial matrix; MMF: Mycophenolate mofetil; PTC: Peritubular capillary; Tac: Tacrolimus; TG: Transplant glomerulopathy; ct: Tubular atrophy.

## Competing interests

All the authors declared no competing interests.

## Authors’ contributions

XH and QS carried out the Clinico-pathological studies, participated in the statistical analysis and drafted the manuscript. CZ participated in the renal pathology studies, SJ participated in the statistical analysis. ZL conceived of the study, and participated in its design and coordination and helped to draft the manuscript. All authors read and approved the final manuscript.

## Authors’ information

Qiquan Sun and Xianghua Huang have contributed equally to the work and are to be considered first authors.

## Pre-publication history

The pre-publication history for this paper can be accessed here:

http://www.biomedcentral.com/1471-2369/13/128/prepub
